# High-quality quartz single crystals for high-energy-resolution inelastic X-ray scattering analyzers

**DOI:** 10.1107/S0021889813004731

**Published:** 2013-06-07

**Authors:** Marcelo Goncalves Hönnicke, Xianrong Huang, Cesar Cusatis, Chaminda Nalaka Koditwuakku, Yong Q. Cai

**Affiliations:** a Universidade Federal da Integração Latino-Americana, Caixa Postal 2044, Foz do Iguacu, Parana 85867-970, Brazil; b Advanced Photon Source, Argonne National Laboratory, Argonne, Illinois 60439, USA; c Departamento de Fisica, Universidade Federal do Parana, Caixa Postal 19091, Curitiba, Parana 81531-990, Brazil; d National Synchrotron Light Source II, Brookhaven National Laboratory, Upton, New York 11973, USA

**Keywords:** X-ray optics, quartz single crystals, spherical analyzers, inelastic X-ray scattering analyzers

## Abstract

Spherical analyzers are well known instruments for inelastic X-ray scattering (IXS) experiments. High-resolution IXS experiments almost always use Si single crystals as monochromators and spherical analyzers. At higher energies (>20 keV) Si shows a high energy resolution (<10 meV), at an exact symmetric back-diffraction condition, since the energy resolution is given by the real part of the susceptibility or polarizability. However, at low energies (<10 keV), high energy resolution is difficult to achieve with Si. α-SiO_2_ (quartz) can be an option, since it offers high energy resolution at low energies. In this work, the characterization of high-quality α-SiO_2_ is presented. Such characterization is made by high-resolution rocking curve, topography and lattice parameter mapping in different samples from a single block. X-ray optics with α-SiO_2_ for IXS at lower energies (from 2.5 to 12.6 keV) with medium to high energy resolution (from 90 to 11 meV) are proposed and theoretically exploited.

## Abstract 2

High-quality quartz (α-SiO_2_) crystals are characterized, and their use for inelastic X-ray scattering analyzers is presented and discussed.

## 1. Introduction

Inelastic X-ray scattering (IXS) is a well defined technique for studying electronic structure in matter. IXS experiments can be resonant (RIXS) and nonresonant (NRIXS). Different interactions can be exploited with IXS, such as core-level excitations, plasmons, valence band excitations, crystal field excitations and phonons. IXS is a technique to be used with synchrotron radiation. Several beamlines for NRIXS are available (Tirao *et al.*, 2004 ▶; Cai *et al.*, 2004 ▶; Hill *et al.*, 2007 ▶), some of them with the use of a multiple element spectrometer to increase the collected scattering angle (Verbeni *et al.*, 2009 ▶). Recently, IXS has also been proposed as a tomography technique with chemical bond contrast (Huotari *et al.*, 2011 ▶). The construction of an IXS spectrometer involves the use of spherical analyzers made, almost always, of high-quality Si or Ge single crystals. These devices are suitable for collecting a reasonable solid angle in order to have enough intensity in the experiment. Also, they work in the back-diffraction geometry, allowing them to achieve high energy resolution. However, depending on the type of interaction that one wants to exploit, the required energy resolution ranges from a few eV to sub-meV. Such requirements are the basics for different construction procedures of spherical analyzers (Verbeni *et al.*, 2005 ▶). The study of phonons almost always requires high to ultra-high energy resolution, in the range of meV to sub-meV. However, high energy resolution can be achieved only at higher energies (>20 keV). The limitation exists because the spherical analyzers work at a back-diffraction condition where the energy resolution is limited by the polarizability (or susceptibility) χ*_h_* of a given crystal. For example, χ*_h_* of Si 555 at 9.89 keV and χ*_h_* of Ge 555 at 9.49 keV are ∼ −1.5 × 10^−6^ and ∼ −3.3 × 10^−6^, respectively. These values limit the energy resolution to ∼15 and ∼30 meV at these energies. Above 20 keV, energy resolution <10 meV can usually be achieved. The interest in IXS at lower energies lies in making new science by studying different kinds of excitations in water, and in organic and biological samples. To circumvent the problem of having no high-energy-resolution monochromators/analyzers at lower energies, alternative geometries have been proposed. The use of Si single crystals and asymmetric back-diffraction has been successfully proposed (Shvyd’ko *et al.*, 2006 ▶; Huang, 2011 ▶; Stetsko *et al.*, 2011 ▶) for ultra-high-energy-resolution IXS. The main limitation of such a setup is the lower acceptance in the analyzer system. This can be slightly increased by using high-angular-acceptance multilayer Montel mirrors (Honnicke, Huang *et al.*, 2010 ▶; Honnicke *et al.*, 2011 ▶). On the other hand, with the advent of high-quality single crystals, such as α-Al_2_O_3_ (sapphire), SiC, GaN and α-SiO_2_ (quartz), new opportunities for X-ray optics have arisen. Sapphire has been used for making IXS spherical analyzers (Yavaş *et al.*, 2007 ▶; Sergueev *et al.*, 2011 ▶). Quartz can be another option, since it offers high energy resolution at lower energies (Sutter *et al.*, 2005 ▶, 2006 ▶). Also, at back-diffraction, noncubic crystalline structures are usually more efficient since multiple beam diffraction effects are minimized. In this work, we will present the structural characterization of high-quality quartz single crystals (X-cut). Such characterization is achieved by using high-resolution rocking curves, topography and lattice parameter mapping in different samples from a single block. X-ray optics for IXS at lower energies (from 2.5 to 12.6 keV) with medium to high energy resolution varying from 90 to 11 meV are proposed and theoretically exploited in both the optical efficiency and energy resolution. Applications using different materials such as Pb- and Lu-based materials are also discussed.

## 2. Energy resolution in back-diffraction

The dynamical theory of X-ray diffraction for the two-beam case (two reciprocal lattice points close to the Ewald sphere) takes into account the interaction between the incident *o* beam and the diffracted *h* beam. In this approximation, outside the crystal, the diffraction process is characterized by two beams, one diffracted in the direction of the incident beam (forward-diffracted *o* beam) and another in the direction of diffraction (diffracted *h* beam). By solving the wave equation in a finite crystal one can find the equations for the diffracted *h*-beam profile (reflectivity curve) as well as the forward-diffracted *o*-beam profile as described elsewhere (Authier, 2001 ▶). For back-diffraction the equations are basically the same; however some of the arguments need to take into account the extended dynamical theory of X-ray diffraction (Caticha & Caticha-Ellis, 1990 ▶). To calculate the *h*-beam and *o*-beam profiles one needs to calculate the polarizabilities (χ_0_ and χ*_h_*), which are obtained from scattering factors calculated by the formalism proposed by Cromer & Liberman (1970 ▶).

**Figure 1:**
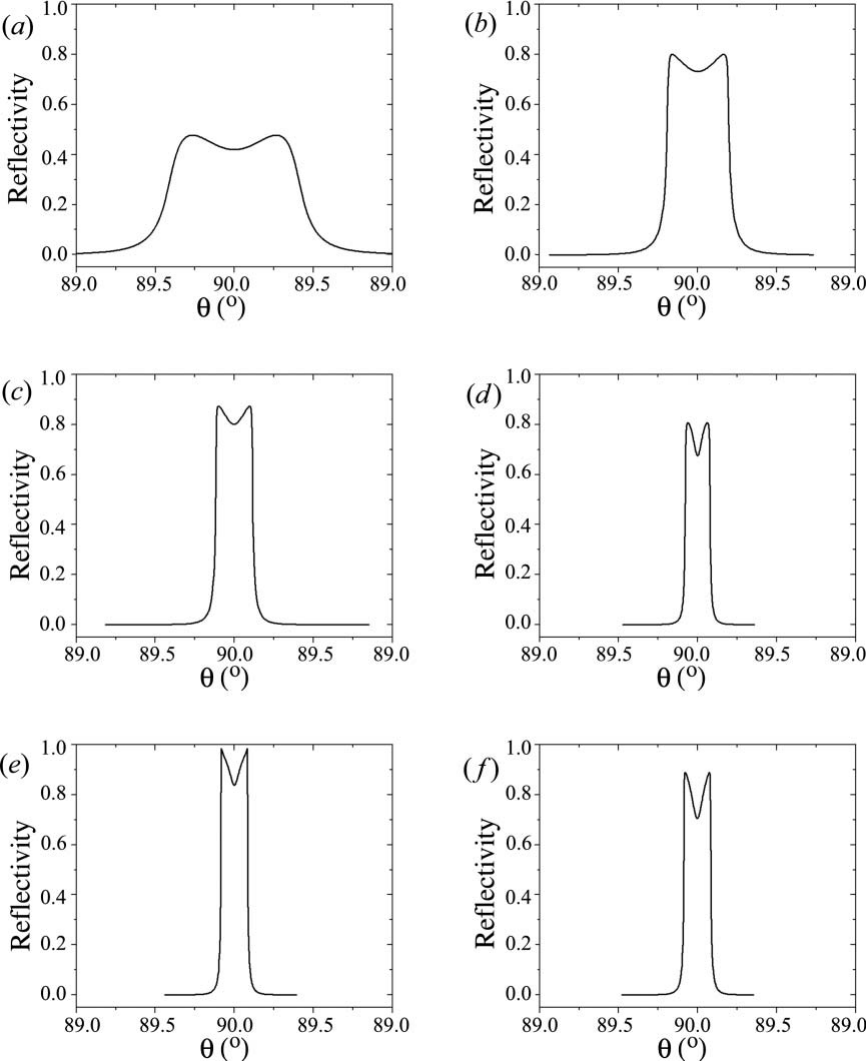
Theoretical symmetric back-diffraction profiles for α-SiO_2_ (quartz) showing high reflectivities even at lower energies. (*a*) α-SiO_2_ 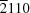 (X-cut) at 2.5 keV, (*b*) α-SiO_2_ 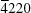 (X-cut) at 5.05 keV, (*c*) α-SiO_2_ 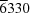 (X-cut) at 7.57 keV, (*d*) α-SiO_2_ 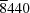 (X-cut) at 10.09 keV, (*e*) α-SiO_2_ 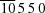 (X-cut) at 12.6 keV and (*f*) α-SiO_2_ 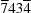 at 9.978 keV.

The calculated diffracted *h*-beam profiles as a function of θ_0_ (diffraction angle) are shown in Fig. 1 ▶ for different energies and different reflections for α-SiO_2_. These are typical back-diffraction *h*-beam profiles for a plane and monochromatic X-ray wave beam. To design an experimental IXS setup by means of which one may approach the theoretical results, we need to study the energy resolution of a monochromator/analyzer in back-diffraction geometry.

To estimate the energy resolution in back-diffraction geometry, we need to have a close look at the general form of Bragg’s law that is valid for conventional and back-diffraction geometries. The equation is found by solving the wave equation in the dynamical theory of X-ray diffraction (Authier, 2001 ▶), and includes the refraction correction and the width of the reflectivity curve:
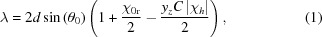
where *y_z_* is the real part of the angular variable *y* (often used in the dynamical theory of X-ray diffraction) and χ_0r_ is the real part of χ_0_. Differentiating equation (1), we can take the general form of the energy resolution for the symmetric Bragg case:
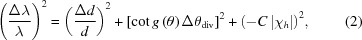
where θ is the Bragg angle and Δθ_div_ is the angular acceptance. If one looks at a DuMond diagram (DuMond, 1937 ▶) it seems that, for exact back-diffraction, the energy resolution is infinitely small. However, by looking at equation (2) one can see that if the crystal is perfect (Δ*d/d* ≃ 0) and it is at back-diffraction (θ = π/2) the energy resolution has a limit given by |χ*_h_*|. For example, for α-SiO_2_ 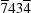 (Sutter *et al.*, 2005 ▶, 2006 ▶) in exact back-diffraction at 9.98 keV, Δλ/λ = Δ*E*/*E* = |χ*_h_*| = 3.5 × 10^−7^ (Δ*E =* 3.5 meV). Then, a monochromator with an energy resolution close to this value is required in order to achieve an energy resolution close to 3.5 meV. Other values of energy resolution at different energies for α-SiO_2_ are shown in Table 1 ▶.

For a setup based on the Rowland circle geometry, as in the most conventional IXS spectrometers, other parameters need to be taken into account in order to estimate the practical energy resolution. Among them are the source size, sample thickness (to be analyzed by IXS), crystallite size of the spherical analyzer, crystal stress (which comes from the spherical analyzer construction process for low- and medium-energy-resolution spectrometers), Johann aberrations (which can be negligible for exact back-diffraction) and deviations from the Rowland circle geometry (Tirao *et al.*, 2004 ▶; Verbeni *et al.*, 2009 ▶).

## 3. Crystal characterization

**Figure 2:**
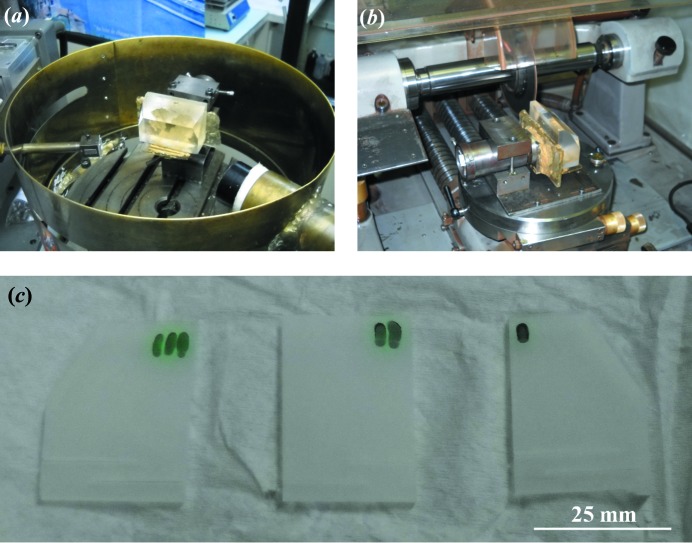
Crystal preparation. (*a*) Quartz block in the orientation camera, (*b*) quartz block for cutting, after orientation, and (*c*) quartz plates over white paper, ready for characterization, after the chemical etching.

**Figure 3:**
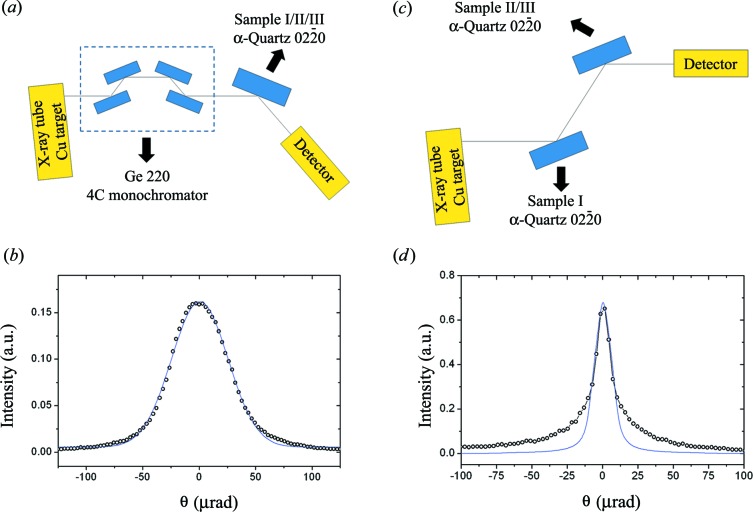
Experimental setup for the rocking curve measurements on α-SiO_2_ crystals. (*a*) Dispersive setup using a Ge 220 four-crystal monochromator (4C), (*b*) α-SiO_2_ 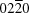 (Y-cut) dispersive rocking curve, (*c*) high-sensitivity nondispersive setup and (*d*) α-SiO_2_ 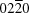 (Y-cut) nondispersive rocking curve. Open circles are the measured data; solid lines are the theoretical calculation based on the dynamical theory of X-ray diffraction. There is an agreement between measured and theoretical data. The major difference is on the tails of the nondispersive rocking curve. This can be attributed to point defects and/or bad surface quality which enhance the ultra-small-angle X-ray scattering.

**Figure 4:**
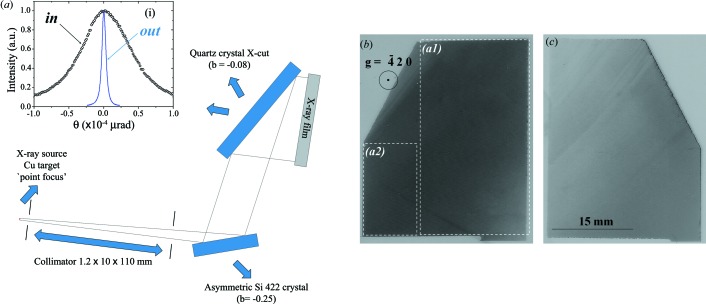
(*a*) Experimental setup for double-crystal X-ray topography of the α-SiO_2_ 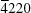 (X-cut) single crystals. In the inset (i) is shown the measured incoming rocking curve (in) and the estimated outgoing rocking curve (out) based on the asymmetry *b* factor. (*b*) and (*c*) show the X-cut double crystal topographies taken on the two different samples. The images shown are homogeneous except for the cutting blade scratches. Dashed areas *a*1 and *a*2 are the regions where the lattice space maps were acquired.

**Figure 5:**
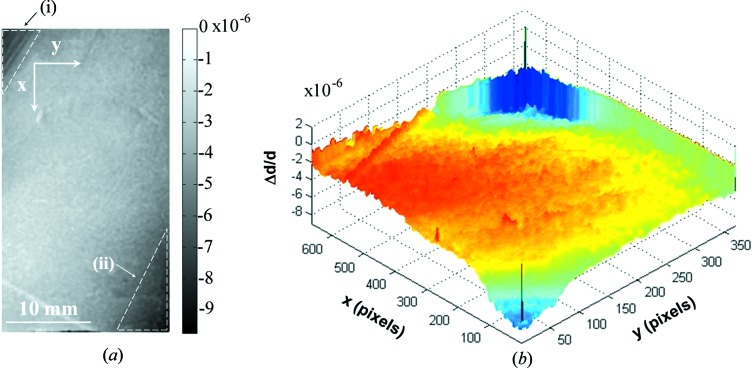
Variations in the lattice parameter map (Δ*d/d* map) of the α-SiO_2_ 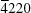 (X-cut) crystal. The map was taken on the dashed area a1 shown in Fig. 4 ▶(*b*). Dashed triangles in (*a*) are the areas where there is a surface flatness: (i) wedge area (due to the orientation flats provided by the manufacturer) and (ii) thickness variation area mostly due to the non-homogeneous etching procedure

Quartz crystals have been under investigation for a long a time (Lang & Miuscov, 1967 ▶; Härtwig *et al.*, 1977 ▶; Yoshimura *et al.*, 1979 ▶; Hönnicke *et al.*, 2004 ▶). Among others, they can be used as piezoelectric sensors in both longitudinal (X-cut crystals) and transverse mode (Y-cut crystals). One application in X-ray optics is their use as a direct stroboscopic X-ray monochromator (Fox & Carr, 1931 ▶; Haruta, 1967 ▶; Zarka * et al.*, 1988 ▶), and they have also been used as an ultrasound inducer for stroboscopic Si monochromators (Hönnicke *et al.*, 2008 ▶) for time-resolved experiments. The synthetic quartz crystal used in the present work was acquired from TEW Japan. The crystal is a grade A sample in terms of quality (*Q*) factor. It was purchased as a single block of 240 × 79 × 40 mm. To characterize it in terms of variations in the lattice parameter (Δ*d/d*), which are important for X-ray monochromators/analyzers, we carried out several different experiments. First of all, we oriented the crystal block and cut it in Y plates 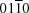 (Fig. 2 ▶). To avoid mounting stress strain relief was included in the crystals for diffraction and topography measurements. After cutting, the crystal was etched in a 40% HF solution. The crystals were not submitted to mechanical polishing. With the crystals ready, dispersive rocking curves with a Ge 220 four-crystal monochromator at Cu *K*α_1_ (8.048 keV) were first carried out (Fig. 3 ▶ *a*) on the α-SiO_2_ 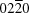 (Y-cut) crystals. Such a setup has sufficient resolution to show variations in the lattice parameter of Δ*d/d* ≃ 1 × 10^−4^. The measured rocking curves (open circles in Fig. 3 ▶ *b*) matched very well with the rocking curves predicted by the dynamical theory of X-ray diffraction (Authier, 2001 ▶) for perfect crystals (solid line in Fig. 3 ▶ *b*). However, we need a more sensitive setup to characterize such a crystal. A nondispersive setup (Fig. 3 ▶ *c*), based on two α-SiO_2_ 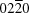 (Y-cut) crystals, is the most suitable since it is strongly sensitive to the variations in the lattice parameter. At 8.048 keV, the variations in the lattice parameter with this setup are Δ*d/d* ≃ 1 × 10^−5^. The measured result is shown in Fig. 3 ▶(*d*) along with the theoretical result predicted by the dynamical theory of X-ray diffraction for perfect crystals. There is a disagreement between the measured and the theoretical rocking curves, especially on the tails of the rocking curve. This can be attributed to the ultra-small-angle X-ray scattering from the not well prepared surfaces as well as point defects. The rocking curve measurements have a limitation owing to the integration of the diffracted intensity in a small area of the crystal. To work out the real reason for the raised tails, a more general characterization was carried out using a double-crystal topography setup. The setup is shown in Fig. 4 ▶(*a*). A long collimator and an asymmetric Si 422 monochromator were used to select Cu *K*β (8.905 keV) and improve the sensitivity in the lattice parameter variation. The topography was taken by also choosing the asymmetric diffraction plane α-SiO_2_ 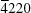 (X-cut). The reason for that was to improve the sensitivity since the outgoing rocking curve is much narrower than the incoming rocking curve. Also, the footprint with the asymmetric reflection covers the entire crystal surface, which helps us to characterize the diffraction plane (α-SiO_2_ 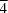
220) that will be used for the spherical analyzer. With this topography setup, variations in the lattice parameter Δ*d/d* ≃ 5 × 10^−7^ could be detected. The topographies are shown in Figs. 4(*b*) and 4(*c*) and are seen to be homogeneous over 79 × 32 mm. Some scratches can be seen on the topography of Fig. 4 ▶(*c*). A variation in the lattice parameter map (Δ*d/d*) was carried out based on these topography images (Hönnicke, Mazzaro *et al.*, 2010 ▶). As an example, the lattice parameter maps of the dashed areas in Fig. 4 ▶(*b*) are shown in Figs. 5 ▶ and 6 ▶. We find that the major variations in the lattice parameter are Δ*d/d* ≃ 5 × 10^−7^ over a large area. There are some peaks of the order of 10^−6^ which can be found close to the crystal edges. In fact, these variations close to the edges are not lattice parameter variations. The intensity variations close to the edges are due to the different crystal thickness in those areas (the crystal surface is not flat). The reasons for that are twofold: (*a*) in the top left of Figs. 5 ▶(*a*) and 6 ▶(*a*) there is a wedge (i) in the crystal, which arises from the crystal block orientation flats provided by the manufacturer, and (*b*) in the bottom right of Fig. 5 ▶(*a*) the crystal surface is not flat (ii), probably as a result of the etching procedure which was not homogeneous, thus producing different thicknesses of the crystal. The lattice parameter maps of the other crystal areas show very similar results. From these results we can say that these quartz crystals can be used for X-ray optics (monochromators and analyzers) to work in the meV range at energies <10 keV. This makes such a crystal suitable for multi-energy inelastic X-ray scattering experiments (RIXS and NRIXS) at lower energies and at high, medium and low energy resolutions.

## 4. Multi-energy and meV-energy-resolution IXS

**Figure 7:**
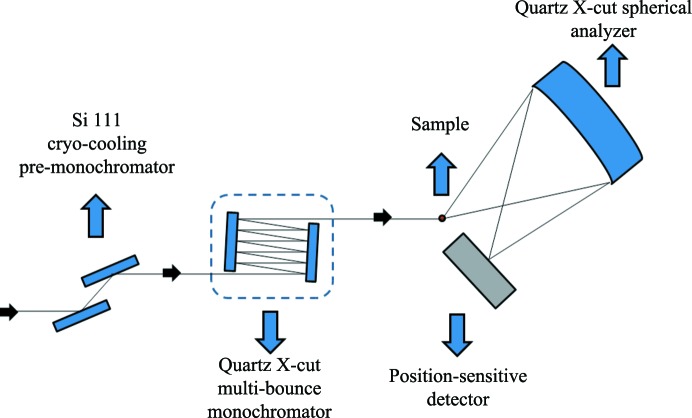
Proposed multi-energy inelastic X-ray scattering spectrometer based on different diffraction orders of the α-SiO_2_ X-cut monochromator and spherical analyzer. The multi-bounce α-SiO_2_ X-cut monochromator is set, in an oven, downstream of an Si 111 cryo-cooling pre-monochromator. The energy scan, based on the inverse geometry, is taken by changing the temperature of the α-SiO_2_ X-cut monochromator. A spherical analyzer, or a set of them, and a position-sensitive detector complete the setup.

**Figure 8:**
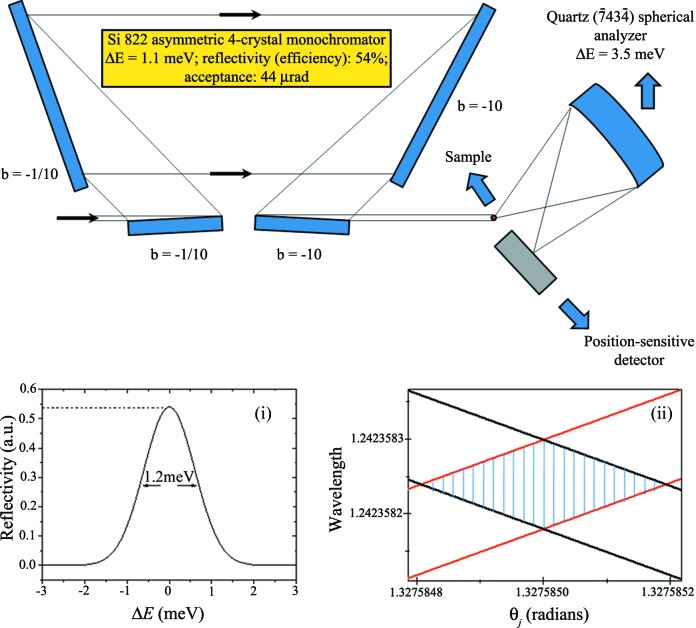
Proposed high-energy-resolution inelastic X-ray scattering spectrometer for studies of phonon excitations. Inset (i) shows the theoretical energy resolution of a four-crystal asymmetric monochromator pointing out the expected optical efficiency (reflectivity) and inset (ii) shows a DuMond diagram of a four-crystal asymmetric monochromator where the divergence and chromaticity can be extracted.

With α-SiO_2_ characterized with very low variations in the lattice parameter, we propose its use as a multi-energy IXS spectrometer as shown in Fig. 7 ▶. Downstream of a cryo-cooling Si 111 pre-monochromator, a multi-bounce α-SiO_2_ X-cut monochromator, to be used at different diffraction orders, is set in an oven in order to perform an energy scan based on the temperature variation of the crystal. The advantage of this type of scan with α-SiO_2_ compared with Si is in the thermal expansion coefficient of α-SiO_2_, which is one order of magnitude greater than that of Si. The α-SiO_2_ X-cut spherical analyzer or set of spherical analyzers is fixed. In this way, the energy scans are performed in the inverse geometry, *i.e.* the incident photon energy is scanned and the scattering is observed at fixed photon energy. A multi-strip detector, for direct observation of the energy dispersion in the focal spot, completes the setup. The calculated energy resolutions and reflectivities for the different energies and different diffraction orders are shown in Table 1 ▶. The reflectivities are shown in order to give an idea of the optical efficiency of this setup. This setup can be used in a variety of IXS experiments (RIXS and NRIXS). As previously mentioned, for practical use, the energy resolution also needs to take into account other geometrical parameters described in §2. For RIXS, the proposed spectrometer can be used, for example, to study different materials based on Lu and Pb. Lu is used in catalysis. Lead phthalocyanine is a Pb-based material used in photovoltaic cells. Since Pb *L*β_1_ ≃ 12.6 keV and Lu *L*α_1_ ≃ 7.6 keV these could be a few of the applications. For NRIXS there are several applications. Among them, X-ray Raman scattering and tomography with chemical-bond contrast can be exploited (Huotari *et al.*, 2011 ▶). To study phonons, a high-energy-resolution setup can be used as shown in Fig. 8 ▶. In this case, an asymmetric four-crystal monochromator (energy resolution ∼1 meV) and optical efficiency of 54% can be applied. As an analyzer α-SiO_2_ 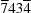 (Sutter *et al.*, 2005 ▶, 2006 ▶) can be used with an energy resolution of 3.5 meV at 9.8 keV and a theoretical reflectivity of 60%. Both spectrometers can be implemented in the new third-generation Brazilian Synchrotron Source (Sirius) and National Synchrotron Light Source II (NSLS II).

## 5. Conclusion

We characterize a different type of crystal to be used as a monochromator and an analyzer for a variety of inelastic X-ray scattering techniques. The grade A (*Q* factor) α-SiO_2_ single crystal shows high quality in terms of variations in the lattice parameter Δ*d*/*d* ≃ 5 × 10^−7^ in an area of 79 × 32 mm. This makes such a crystal affordable for making spherical analyzers, as well as X-ray monochromators, for energy resolution of the order of meV.

## Figures and Tables

**Figure 6:**
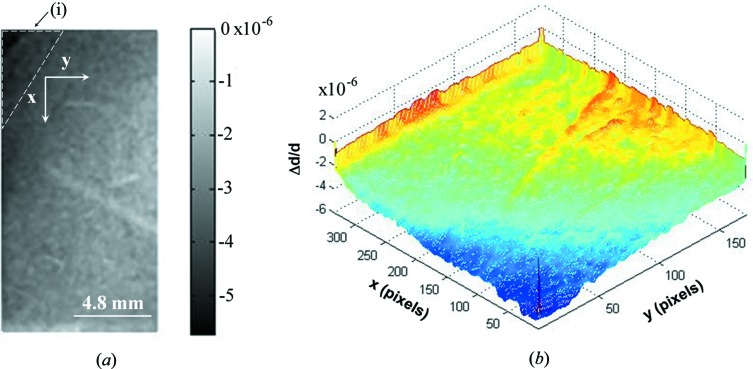
Variations in the lattice parameter map (Δ*d/d* map) of the α-SiO_2_ 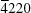 (X-cut) crystal. The map was taken on the dashed area *a*2 shown in Fig. 4 ▶(*c*). The dashed triangle in (*a*) is the wedge area (i) due to the orientation flats provided by the manufacturer.

**Table 1:** Reflections, back-diffraction energies, energy resolution and theoretical reflectivities for the multi-energy IXS α-SiO_2_ (quartz) spherical analyzer

Reflection	Energy(eV)	Energy resolution(meV)	Theoreticalreflectivity
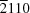 (X-cut)	2523.82	90	0.47
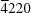 (X-cut)	5047.30	51	0.80
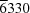 (X-cut)	7570.86	23	0.87
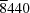 (X-cut)	10094.44	11	0.80
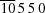 (X-cut)	12618.2	15	0.98
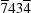 †	9979.84	3.5	0.60

† Already proposed and characterized by Sutter *et al.* (2005 ▶).
